# Solitary fibrous tumors from A to Z: a pictorial review with radiologic-pathologic correlation

**DOI:** 10.1186/s13244-025-01991-x

**Published:** 2025-05-28

**Authors:** Fatmaelzahraa Abdelfattah Denewar, Mitsuru Takeuchi, Doaa Khedr, Fatma Mohamed Sherif, Farah A. Shokeir, Misugi Urano, Ahmed E. Eladl

**Affiliations:** 1 Department of Radiology, Faculty of Medicine, Mansoura University, Mansoura, Egypt; 2 Department of Radiology, Radiolonet Tokai, Nagoya, Japan; 3 Department of Radiology, Kawasaki Medical School, Kurashiki, Japan; 4 Department of Radiology, Nagoya City University Graduate School of Medical Sciences, Nagoya, Japan; 5 Department of Pathology, Faculty of Medicine, Mansoura University, Mansoura, Egypt

**Keywords:** Solitary fibrous tumor, Hemangiopericytoma, Magnetic resonance imaging, Computed tomography

## Abstract

**Abstract:**

Solitary fibrous tumors (SFTs) represent a rare subset of mesenchymal neoplasms, affecting 1–2 per million people, with no gender preference. They demonstrate indolent behavior, frequent asymptomatic presentation, and widespread anatomical involvement. At imaging, SFTs typically appear as well-defined, predominantly hypervascular masses with varying degrees of cystic change and necrosis, though calcification is rare. Avid heterogeneous enhancement is typical following intravenous contrast administration, with multiple blood vessels observed at the periphery. Although findings on CT and MRI alone are generally nonspecific, a frequent feature of SFTs at MRI is the presence of rounded or linear low signal intensity foci on T1- and T2-weighted images, corresponding to the fibrous and collagenous content. Nevertheless, because the imaging features of SFTs overlap with those of many benign and malignant tumors, histologic confirmation is required for the final diagnosis. A comprehensive understanding of SFTs’ multifaceted clinical, pathological, and radiological presentations across various organs is crucial for accurate diagnosis and effective management.

**Critical relevance statement:**

A comprehensive understanding of the classic radiological and pathological features of solitary fibrous tumors across various organs is crucial for accurate diagnosis and effective management.

**Key Points:**

- Solitary fibrous tumors (SFTs) are rare hypervascular fibrous tumors with indolent behavior.
- Imaging features of SFTs overlap with many other tumors, necessitating histologic confirmation.
- Understanding SFTs’ radiological presentations is crucial for accurate diagnosis and effective management.

**Graphical Abstract:**

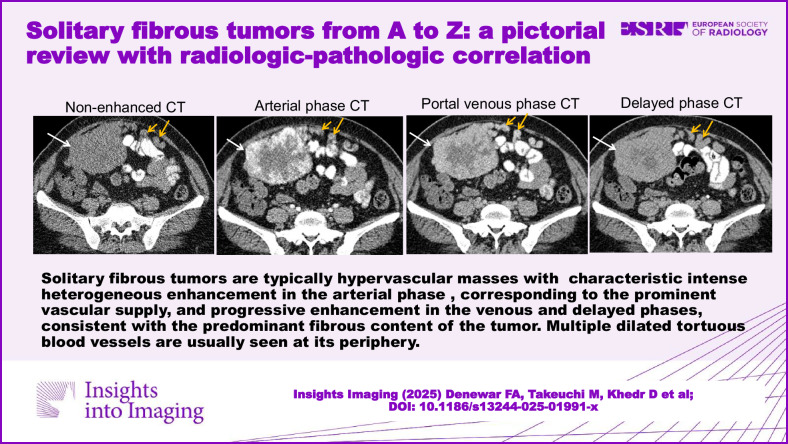

## Introduction

Solitary fibrous tumors (SFTs) are rare fibroblastic mesenchymal neoplasms with an incidence rate of 1–2 per million and no gender predilection [1, 2]. SFTs characteristically demonstrate slow growth, frequently resulting in asymptomatic presentation. Their anatomical distribution encompasses central nervous system (CNS) and non-CNS sites, with pleural involvement comprising 30% of cases, followed by meningeal (27%), abdominal/pelvic (20%), and other locations [3, 4]. While many SFTs are benign, about 10–30% show aggressive or malignant behavior such as local invasion, distant metastases, and recurrence [5–7]. A distinctive feature of SFTs is the fusion of NAB2 (EGR1-binding protein 2) gene and signal transducer and activator of transcription 6 (STAT6) gene, both located on chromosome 12q13, as NAB2-STAT6 fusion gene. STAT6 immunohistochemistry (IHC) positivity is a good marker for the presence of these fusions [8, 9]. Due to overlapping imaging characteristics with various lesions, histologic confirmation is essential for definitive diagnosis of SFTs. Radiologists play a crucial role in suggesting preoperative diagnosis and guiding optimal treatment strategies. In this article, we present a comprehensive review of SFTs, placing special emphasis on classic imaging features, with pathologic correlation.

## Historical evolution and classification of SFTs

The historical evolution of SFTs traces back to the early 20th century, when Klemperer and Rabin described the morphological features of SFTs in a series of 5 cases in the pleura for the first time in 1931 [10]. Similar tumors were reported by Stout and Murray in 1942 and labeled as “localized mesothelioma of pleura” [11]. However, in 1951, Stout and Himadi renamed these tumors ‘solitary fibrous tumors’ [12]. The first series of extrathoracic SFT was published by Goodlad et al in 1991 [13].

The term “Hemangiopericytoma (HPC)” was used for the first time by Stout and Murray in 1942 while describing a series of 9 cases [11]. The diagnostic criteria for HPC were refined and features for assessment of malignancy were established by Enzinger and Smith in a large study of 106 cases [14]. For many years, HPC and SFTs were considered as two histological subtypes of a single neoplasm due to their clinicopathological similarities [15, 16]. The discovery of a shared unifying molecular signature between both tumors, which is the recurrent fusion of NAB2 and STAT6 genes located at chromosomal region 12q13, has confirmed their identical nature [8, 9]. Thus, both tumors were merged into a single entity in the 4th edition of the World Health Organization (WHO) classification of soft tissue tumors [17]. The recent 2020 WHO classification of tumors of soft tissue and bone has classified SFT as a fibroblastic neoplasm with intermediate (rarely metastasizing) behavior [18]. However, in the current WHO classification of CNS tumors, extrameningeal SFT and HPC are described as a single group, but different histologic grades are assigned to these tumors while retaining the names [19].

Accurate differentiation between CNS and non-CNS SFTs is critical due to variations in clinical behavior and treatment response. The WHO classification schema stratifies CNS SFTs into three grades, considering histopathological features such as mitotic rate and necrosis. Grade 1 (benign), characterized by low mitotic activity and absent necrosis, with favorable prognosis; Grade 2 (atypical), exhibiting moderate mitotic activity and focal necrosis, with increased recurrence risk; and Grade 3 (malignant), featuring high mitotic activity, extensive necrosis and heightened recurrence/metastasis risks [20]. Non-CNS SFTs are classified into benign (locally invasive), NOS (not otherwise specified, rarely metastatic), and malignant based on distinct clinical behaviors and histological characteristics [2]. Benign SFTs exhibit well-circumscribed margins, slow growth, low mitotic activity, and absent necrosis. NOS tumors demonstrate intermediate characteristics, while malignant SFTs display high mitotic activity, pleomorphism, and necrosis, necessitating aggressive treatment [2].

## Gross and microscopic picture

**Fig. 1:**
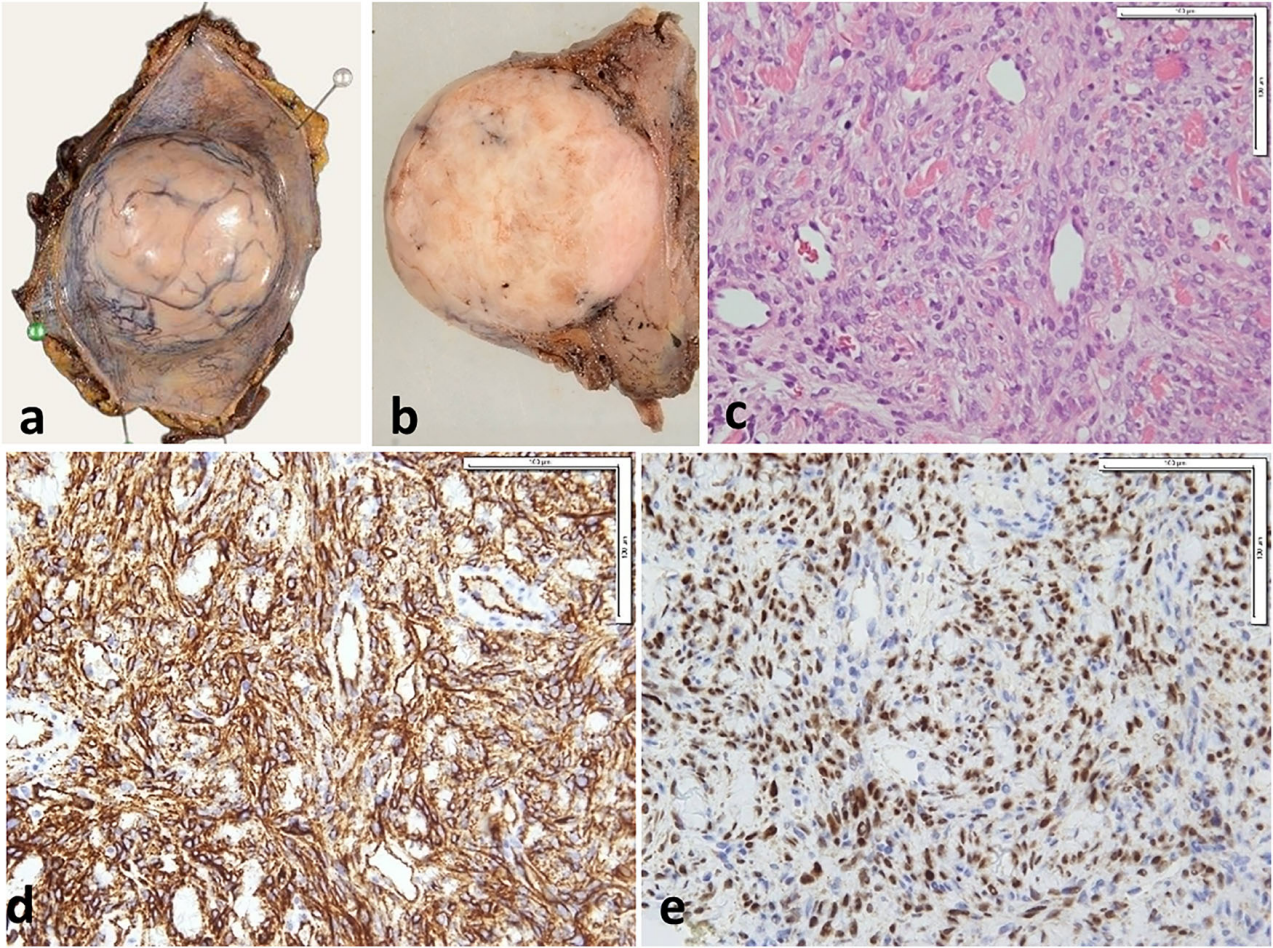
SFT of the anterior abdominal wall in a 40-year-old male. Gross appearance of the surgical specimen (**a**, **b**) shows a circumscribed, rounded mass with abundant blood vessels on its surface (**a**) and solid yellowish-white cut surface (**b**). Microscopic examination (**c**–**e**) demonstrates ovoid to fusiform spindle cells proliferating around dilated blood vessels against a background of collagenous stroma (**c**). The tumor cells are diffusely positive for CD34 (**d**) and STAT6 (**e**)

Grossly, SFTs are well-circumscribed firm tissue masses, variable in size (up to 20 cm or more) and showing homogenous whitish cut section with possible microcystic or hemorrhagic areas [4]. Microscopically, they are characterized by proliferation of haphazardly arranged spindle-shaped cells within variably collagenous stroma and with streaming of tumor cells between collagen fibers (Fig. 1). The stroma often shows characteristic staghorn-like (hemangiopericytoma-like) blood vessels [21]. Cases with myxoid stromal changes were described [22]. A lipomatous variant with increased mature fat content [23] and giant cell-rich variant showing admixed multinucleated giant cells [24] were also described. Malignant SFTs show hypercellularity, mitotic rate (more than 4/10 HPF), areas of necrosis and infiltrative margins [4]. De-differentiated tumors are characterized by abrupt transition to areas of high-grade sarcomatous changes with or without heterologous component [25, 26].

The diagnosis of SFTs has been clarified by molecular features, particularly the characteristic NAB2-STAT6 gene fusion, which can be detected using multiplexed sequencing assays. IHC also plays a key role, with strong nuclear STAT6 staining being a hallmark of SFTs [27, 28]. Additionally, CD34 is a supportive marker, showing strong and diffuse expression in most SFTs (over 80%), although its expression may be lost in more aggressive tumors [28].

## Risk stratification systems

SFTs predominantly exhibit indolent behavior. However, malignant potential is signaled by tumor size, necrosis, pleomorphism, elevated mitotic rates (≥ 4/10 HPF), and dissemination at presentation, necessitating rigorous assessment.

According to the 2020 WHO classification, ‘Typical’ or ‘atypical’ designations are discouraged due to overlap with aggressive lesions. Recent advances have led to the development of risk-stratification models for predicting metastasis occurrence [29, 30]. Demicco et al’s seminal studies [5, 6] established a robust risk assessment model for SFTs, incorporating age, tumor size, mitotic count, and necrosis, facilitating global applicability [5, 6] (Table 1).

**Table 1:** New four-variable risk model for the prediction of metastatic risk of SFTs

Risk factor	Cutoff value	Points assigned
Age (years)	< 55	0
	> 55	1
Mitoses (/10 high-power field)	0	0
	1–3	1
	≥ 4	2
Tumor size (mm)	40	0
	50–99	1
	100–149	2
	≥ 150	3
Tumor necrosis	< 10%	0
	> 10%	1
Risk	Low	0–2 points
	Intermediate	3–4 points
	High	5–6 points

The French Sarcoma Group introduced a prognostic risk calculator for SFTs, integrating patient age, tumor location, mitotic activity, and radiotherapy exposure, highlighting the necessity of prolonged surveillance [7]. Recent investigations have refined SFTs risk stratification. Georgiesh et al [31] demonstrated sex-specific disparities and correlations with mitotic activity/necrosis, whereas Zhang et al [32] introduced an innovative three-tiered model integrating molecular and IHC variables.

Current risk stratification models predominantly cater to non-CNS SFTs, whereas CNS SFTs lack specialized models, relying on the WHO grading system for predicting aggressiveness and recurrence potential [20].

## Clinical features

SFTs primarily affect individuals in their 50s and 60s, with pleural SFTs presenting at older ages [27, 33]. Additionally, tumors in the mediastinum, peritoneum, or retroperitoneum exhibit increased aggressiveness [34]. The clinical presentation depends on tumor location: CNS SFTs present with increased intracranial pressure symptoms, while non-CNS SFTs often remain asymptomatic until reaching 5–8 cm [35–37]. Although SFTs typically exhibit localized growth, a subset of cases can metastasize to distant sites, including the lungs, liver, and bones [38]. Pierre-Marie-Bamberger syndrome, characterized by hypertrophic osteoarthropathy, affects approximately 10% of patients with pleural SFTs and is linked to vascular endothelial growth factor overexpression [39]. Additionally, Doege-Potter Syndrome, a rare hypoglycemic disorder, affects approximately 5% of SFT patients and is attributed to insulin-like growth factor 2 overproduction [40].

## Classic imaging features of SFTs

At imaging, SFTs typically appear as well-defined, predominantly hypervascular masses with varying degrees of cystic change and necrosis, though calcification is rare. They typically demonstrate avid heterogeneous enhancement following intravenous contrast administration. Multiple peripheral blood vessels are observed at the periphery [33].

Ultrasound can be used in specific instances of SFTs based on their location. It is useful in superficial SFTs such as orbital, extremities, as well as large lesions in the abdominopelvic cavity and peripheral intrathoracic lesions [41]. Ultrasound findings vary from hypoechoic and homogeneous in small lesions to heterogeneous in larger and malignant ones due to central degeneration [33]. SFTs’ characteristic staghorn-like vessels often have low-velocity flow, potentially limiting color Doppler detection [42].

**Fig. 2:**
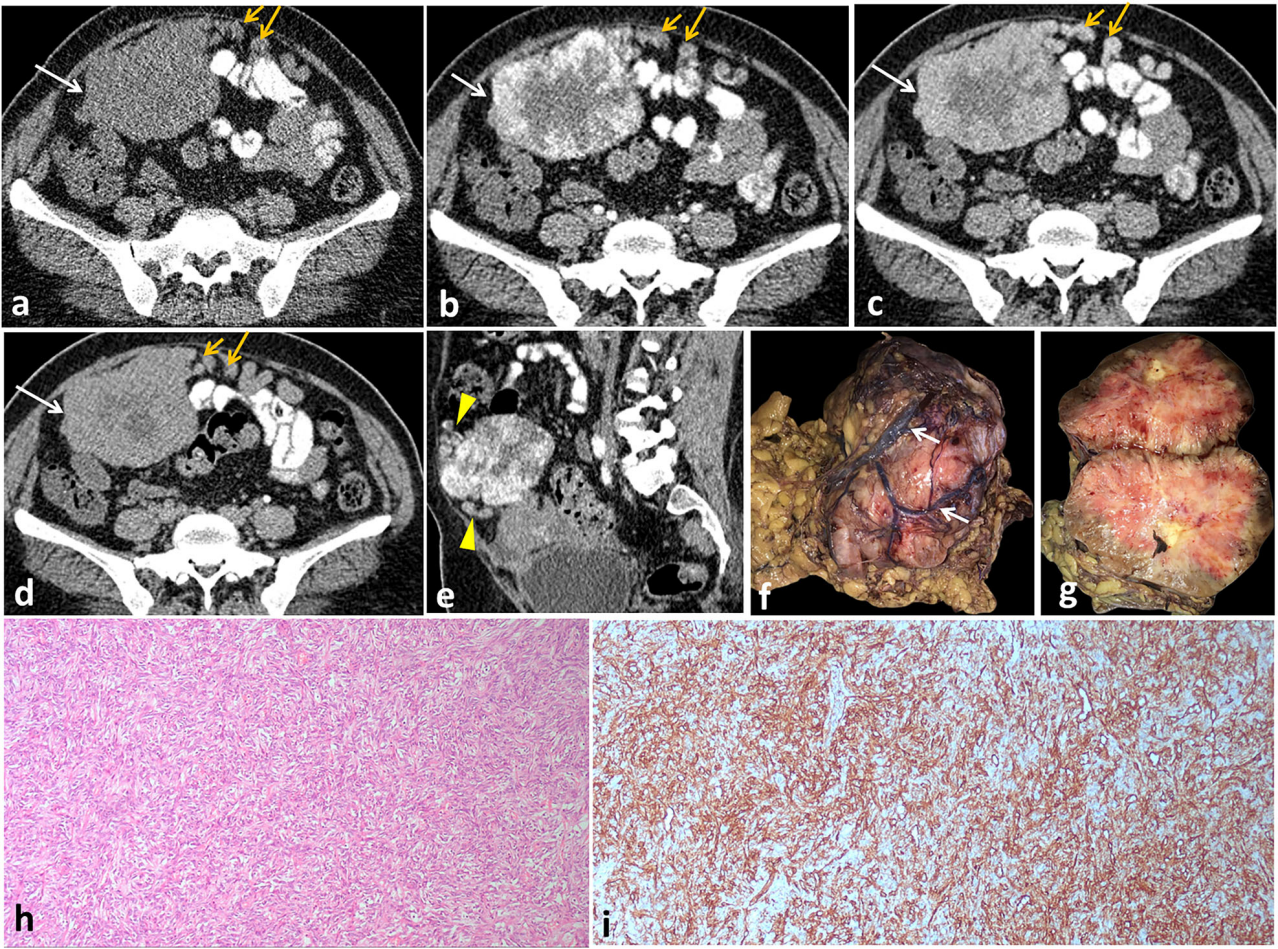
SFT of the mesentery in a 46-year-old female with diarrhea for 1 month. Non-contrast (**a**) and contrast-enhanced axial CT images in arterial (**b**), portal venous (**c**), and delayed (**d**) phases showing a sharply demarcated lobulated hypervascular mesenteric soft tissue mass seen contacting anterior abdominal wall muscles and displacing surrounding small bowel loops. The mass shows homogeneous isodensity to muscles in non-contrast CT (arrows in **a**) with intense heterogeneous enhancement in the arterial phase (arrows in **b**), corresponding to the prominent vascular supply, and progressive enhancement in the venous and delayed phases (arrows in **c** and **d**), consistent with the predominant fibrous content of the tumor. Multiple dilated tortuous blood vessels are seen at its periphery (yellow arrows), arising from the basin of the splenic vessels. Sagittal reformatted image in the arterial phase (**e**) shows the mass with multiple tortuous blood vessels seen at its peripheral parts (arrowheads). Grossly (**f**, **g**), it appears as a firm, well-circumscribed, lobulated, non-capsulated solid tumor with tan to reddish colored cut section. Prominent tumor vascularity is seen on the surface (white arrow in **f**). Microscopic examination reveals fusiform spindle-shaped tumor cells arranged in short, ill-defined fascicles within a collagenized background (**h**) with CD34 diffusely positive tumor cells (**i**)

**Fig. 3:**
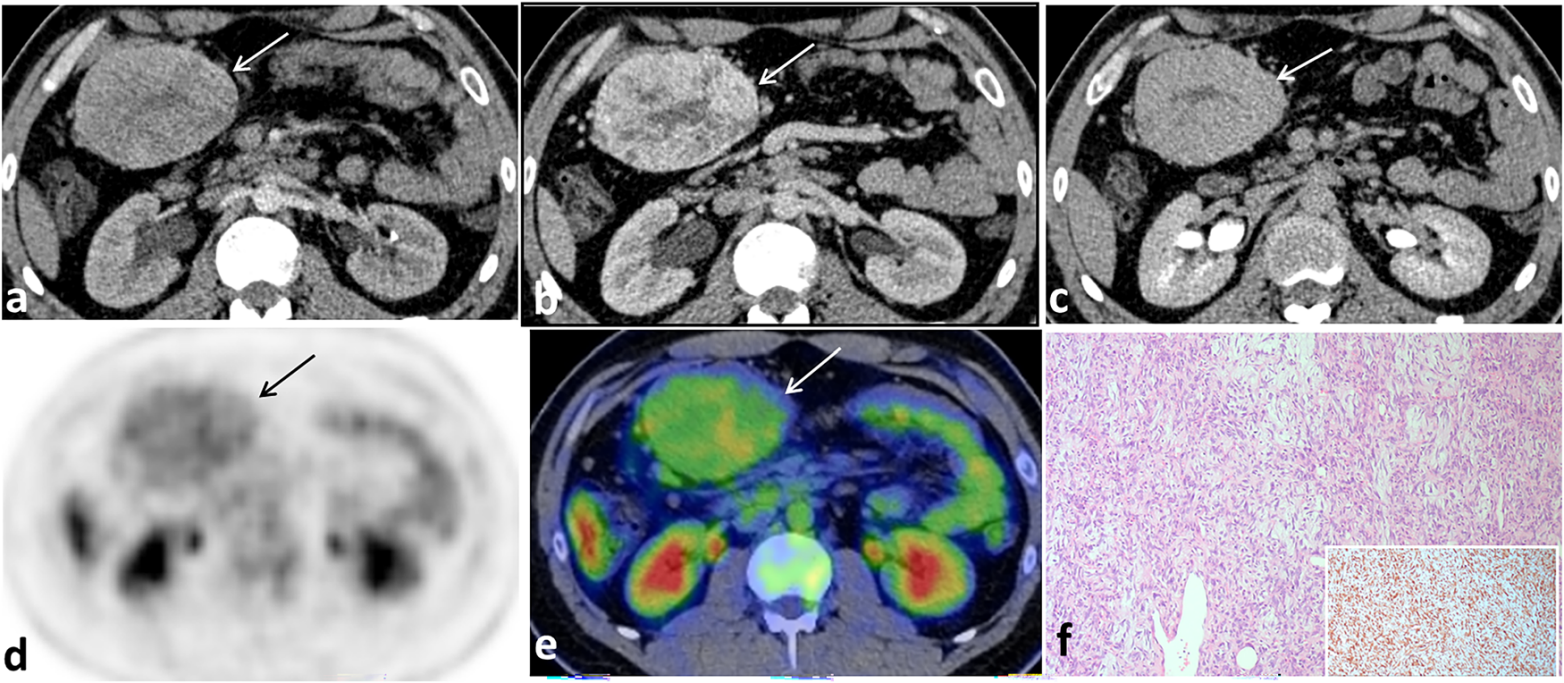
SFT of the mesentery in a 28-year-old male. Contrast-enhanced axial CT images in arterial (**a**), portal venous (**b**), and delayed (**c**) phases showing a well-defined lobulated hypervascular mesenteric soft tissue mass at the right lumbar region, and displacing surrounding small bowel loops. The mass demonstrates heterogeneous enhancement in the arterial phase (arrow in **a**) with progressive enhancement in the venous and delayed phases (arrows in **b**, **c**), consistent with the predominant fibrous content of the tumor. Axial 18F-FDG-PET (**d**) and PET/CT fused images (**e**) reveal moderately FDG-avid mesenteric mass. Microscopically (**f**), haphazardly arranged fusiform spindle tumor cells with dilated staghorn-like blood vessels are seen. Tumor cells are diffusely positive for STAT-6 (inset)

Computed tomography (CT) and magnetic resonance imaging (MRI) are the modalities of choice for the detection of the tumor, its local extension and invasion into adjacent structures, and locoregional or distant metastases. CT and MRI images of SFTs reflect their histopathologic characteristics. On non-enhanced CT, SFTs appear as solid masses of intermediate to high attenuation in regions with high cellularity and fibrous stroma, whereas necrotic or cystic regions show lower attenuation. Calcifications are rare and can be seen in large-sized tumors [33, 36, 43, 44]. As hypervascular tumors, SFTs characteristically demonstrate intense, heterogeneous enhancement during the early arterial phase of dynamic contrast-enhanced CT, attributed to the presence of dilated, staghorn-shaped vessels. Progressive enhancement is observed in later phases, corresponding to hypervascular and hypercellular regions on histopathological examination. Collagenous or fibrotic stroma gradually accumulates contrast, exhibiting delayed enhancement (Figs. 2, 3). However, necrotic or degenerated cystic areas do not show enhancement. Avid contrast enhancement is seen in 65% of cases, and large collateral feeding vessels are verified in 35% of cases [45].

**Fig. 4:**
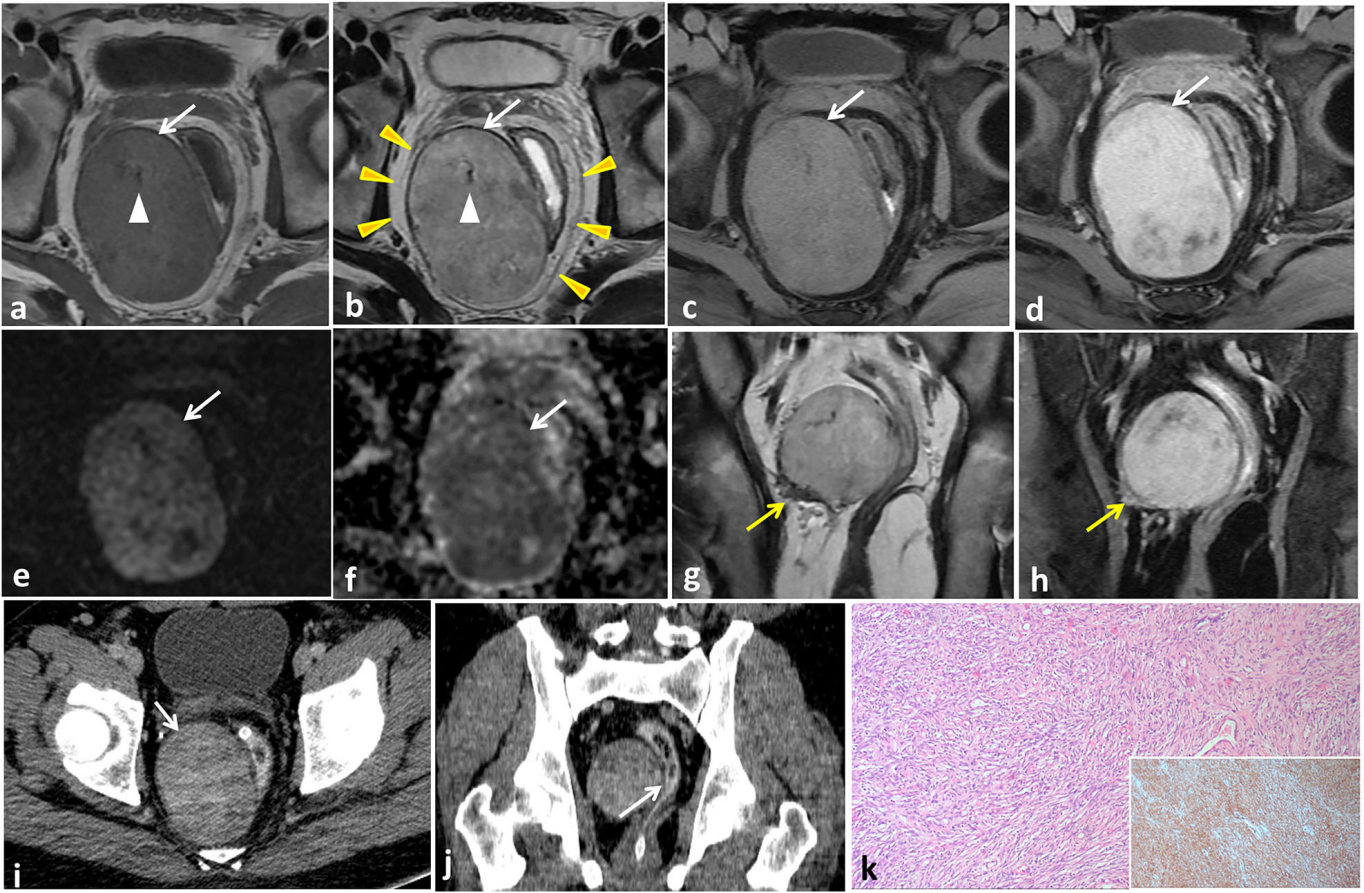
SFT of the mesorectum in a 42-year-old male with lower abdominal pain. Axial T1- (**a**) and T2- (**b**) weighted MR images show a well-defined oval mass at the right lateral aspect of the rectum compressing its middle third (arrows). The mass is seen completely confined within the mesorectal fat and enclosed by the mesorectal fascia (arrowheads). It displays intermediate T1 and mixed T2 signal intensity with outer low signal rim and low signal streaks within. Axial gadolinium-enhanced fat-suppressed T1-weighted MRI in early (**c**) and late (**d**) phases shows mild early enhancement of the mass with marked progressive late heterogeneous enhancement (arrows). Axial diffusion-weighted MRI with high b-value (**e**) and corresponding ADC map (**f**) show restricted diffusion and low ADC value of the mass (arrows). Coronal T2- (**g**) and gadolinium-enhanced fat-suppressed T1- (**h**) weighted MR images show extra-tumoral flow voids and dilated vessels (arrows). Axial (**i**) and coronal reformatted (**j**) post-contrast CT images show heterogeneous enhancement of the mass with clear fat planes between it and the rectum (arrow in **j**). Microscopic examination (**k**) and CD34 (inset) show intersecting fascicles of spindle tumor cells with collagen deposition. Diffuse positive CD34 reaction is seen in tumor cells

On MRI, SFTs typically reveal intermediate signal intensity on T1-weighted image (WI) and heterogeneous signal intensity on T2-WI, reflecting varying tissue composition, with areas of low signal (collagen/fibrosis), intermediate signal (cellular areas), and high signal (myxoid areas) [33, 42]. Streaks of low signal intensity foci on T1- and T2-weighted images are frequently observed within the tumor, corresponding to its fibrous and collagenous components (Fig. 4) [42]. Gadolinium-enhanced imaging reveals intense enhancement, often accompanied by central non-enhancing areas and serpentine peripheral vessels. Enhancement patterns correlate with tumor composition: hypercellular areas show persistent/prolonged venous phase enhancement, whereas fibrous/collagenous stroma exhibits mild arterial phase enhancement with delayed phase intensification [33, 36, 46, 47].

Advanced MRI techniques, such as diffusion-weighted imaging (DWI) using apparent diffusion coefficient (ADC) values and histogram analysis of ADC maps, have shown promising results in differentiating intracranial SFTs from meningiomas [48]. In addition, dynamic contrast-enhanced (DCE) MRI has demonstrated potential in distinguishing ocular SFTs from schwannomas [49]. Angiographic evaluation of SFTs typically reveals a highly vascularized mass characterized by arborizing vessels originating from a vascular pedicle [36, 47, 50].

The utility of positron emission tomography (PET)/CT in distinguishing benign from malignant SFTs is uncertain, mainly due to the tumor’s rarity and limited available data. Research on FDG uptake in SFTs has yielded conflicting results. Some studies suggest that benign SFTs tend to have low or no FDG uptake, while malignant ones have higher uptake [51, 52]. However, other studies have found overlap in standardized uptake values (SUVs) between benign and malignant lesions, with false-positive and false-negative cases reported [53, 54]. As a result, the utility of SUVs in distinguishing between benign and malignant SFTs remains a topic of debate (Fig. 3).

PET/CT is crucial for evaluating disease spread, detecting distant metastases, and monitoring for recurrence after treatment [54]. Larger studies are necessary to elucidate the relationship between FDG uptake in SFTs and tumor biology, including grade, metastasis, and survival rates [53].

## Imaging spectrum of SFTs in different organs

### CNS

Intracranial SFTs are rare, typically extra-axial and meningeal in origin, with intra-axial cases being exceedingly rare [55]. The patients usually present with headache or other clinical features related to the tumor site, such as seizures and focal neurological deficit [56, 57].

**Fig. 5:**
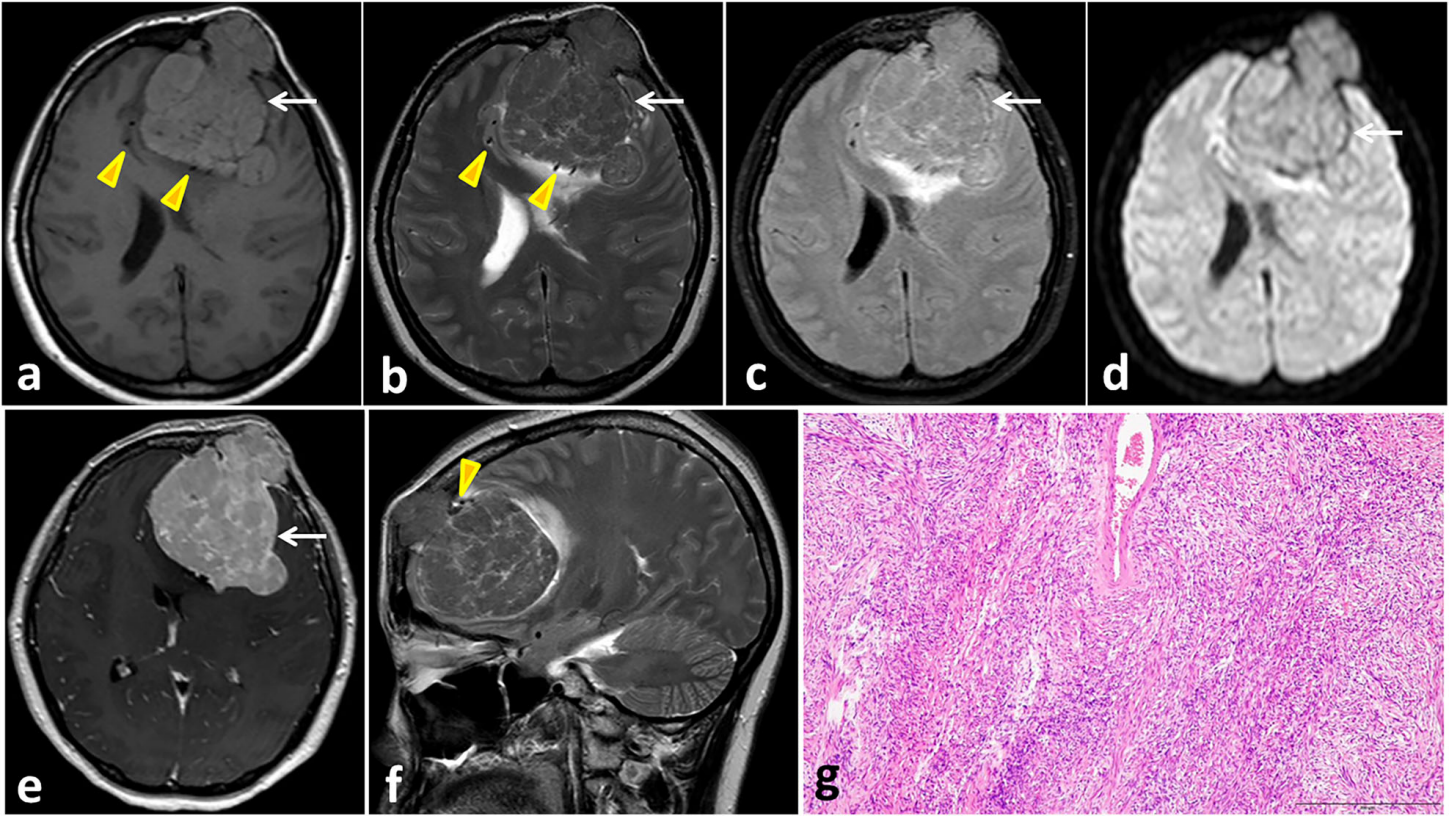
Meningeal SFT in a 44-year-old female with left forehead swelling for 5 years presented with a recent headache. Axial T1- weighted (**a**), T2- weighted (**b**), FLAIR (**c**), DWI b-1000 (**d**), contrast-enhanced T1-weighted (**e**), and sagittal T2-weighted (**f**) MR images show a large left extra-axial space occupying lesion with bone destructive changes and mushrooming (DDx, hemangiopericytoma or atypical meningioma). The mass reveals intermediate T1 signal, heterogeneous intermediate T2 signal, intense but uneven enhancement, and no restricted diffusion (arrows). The mass compresses the left lateral ventricle, causing midline shift, and is surrounded by dilated vessels indicated by serpentine flow voids (arrowheads). Microscopic examination (**g**) shows fusiform spindle-shaped tumor cells running in an intersecting fascicular pattern

At CT, a well-circumscribed, smooth, lobulated iso- to hyperattenuating soft tissue mass is evident, with uncommon scattered calcifications. Smaller tumors tend to enhance homogeneously, whereas larger lesions may have cystic or necrotic change. Associated calvarial erosions may be seen [58, 59]. On MRI, SFTs display relative heterogeneous intermediate signal intensity on both T1- and T2-WIs with possible existence of areas of low fluid attenuated inversion recovery (FLAIR)/T2 signal intensity, due to fibrous tissue. Areas of subarachnoid hemorrhage, cystic changes and necrosis are usually noticed. Regions of collagen can appear as large regions of low signal intensity on T1-WI and T2-WI, giving rise to a ‘Yin-Yang’ shape appearance. On susceptibility-weighted images, SFTs can reveal multiple intralesional signal flow voids [60, 61]. On gadolinium-enhanced study, STFs show intense contrast enhancement with dural tail sign in a small proportion of lesions (Fig. 5). If a central focus of heterogeneity and variable contrast enhancement is exhibited, malignant change should be considered. Grade 3 lesions are more likely to cross the midline with invasion of the adjacent dural venous sinuses, as well as overlying skull and soft tissues [58].

Most of the solid parts of lesions show areas of restricted diffusion. On perfusion-weighted images, SFTs display hyperperfusion with relative cerebral blood volume values ranging from 7 to 7.5. On MR spectroscopy, these lesions show high lipid lactate peaks as well as high myoinositol peak, especially in higher-grade lesions, distinguishing them from meningioma [62, 63].

Due to the high vascularity of SFTs, the lesions reveal prominent tumor blush on digital subtraction angiography (DSA), with varying feeding vessels, according to the tumor location. In addition to meningeal supply, these tumors can receive pial blood supply [58].

Imaging features of SFTs can overlap with other dural-based intracranial masses such as meningioma, dural metastasis, and lymphoma. Meningioma is usually demonstrated as an avidly enhancing extra-axial dural-based mass with an enhancing dural tail. Tumoral calcification and adjacent hyperostosis are characteristic findings. On DWI, SFTs usually exhibit lower ADC values than meningiomas due to their higher cellularity [48]. On MR spectroscopy, a high choline/creatine ratio and low NAA peaks are demonstrated. Also, alanine peak, if present, is characteristic of meningioma [62, 64]. Dural metastases are typically revealed as multiple focal lesions with intense contrast enhancement and enhancing dural tail in about 50% of cases [65]. If diffuse involvement takes place, they appear as smooth dural thickening following the contour of the calvarium [66]. They are usually associated with adjacent bony destruction; however, there may be hyperostosis in the case of osteoblastic prostate metastasis. Direct invasion of brain parenchyma is observed in about one-third of cases [67]. Lymphoma usually demonstrates hyperattenuation on CT images and low signal intensity on T2-WI MRI due to hypercellularity, with intense homogeneous contrast enhancement. Calvarial involvement is uncommon, and flow voids are usually absent. On DWI, the lesions show restricted diffusion. Elevated lipid/lactate peaks and high choline to creatine ratios are depicted on MR spectroscopy [68, 69].

### Spine

Intraspinal SFTs are extremely rare, typically arising from the meninges, nerve roots, or spinal cord parenchyma. Most cases (64%) are intramedullary, while extramedullary, intradural tumors often extend into the spinal cord [70]. Intramedullary tumors usually originate from the pial surface, subpial, or perivascular tissues, and can exhibit a continuum of growth patterns from intramedullary to extramedullary [71].

Differential diagnosis should consider classical extramedullary tumors, such as meningiomas, nerve sheath tumors, and hemangiopericytomas. While most spinal SFTs are benign, they can recur locally, exhibit malignant potential, or metastasize. Imaging characteristics are similar to those at other sites, featuring hypointense T1 signals, variable T2 signals, and variable enhancement [72].

## Head and neck

### Orbit

SFTs frequently occur in the orbit, accounting for 6–25% of head and neck cases. Orbital SFTs can manifest as intraconal or extraconal lesions, sometimes affecting the lacrimal sac or eyelid. Clinical symptoms are typically nonspecific and compressive, including eye swelling, proptosis, and visual disturbances, without systemic symptoms [73]. Imaging characteristics are often nonspecific, showing a slowly growing mass with avid enhancement on CT and MRI. Dual-phase CT and DCE MRI can reveal rapid enhancement and early washout, while CT angiography can demonstrate the tumor’s rich vascular supply [49]. Osseous erosions of the orbital wall should raise concern for malignant potential [74].

When diagnosing orbital SFTs, it’s essential to distinguish them from other similar lesions such as cavernous hemangiomas and schwannomas. Cavernous hemangiomas are characterized by hyperintensity on T2-weighted MRI with low signal fibrous septa and phleboliths, as well as distinct enhancing features that exhibit delayed pooling of contrast material on dynamic investigations, which helps distinguish them from SFTs [74]. Schwannomas typically exhibit T2 hyperintensity with moderate enhancement on contrast-enhanced images. DCE MRI can help differentiate SFTs from schwannomas, as SFTs often exhibit a washout curve due to their high cellularity, whereas schwannomas tend to display persistent or plateau-shaped curves due to their loose cellular arrangement [49].

### Paranasal sinuses

**Fig. 6:**
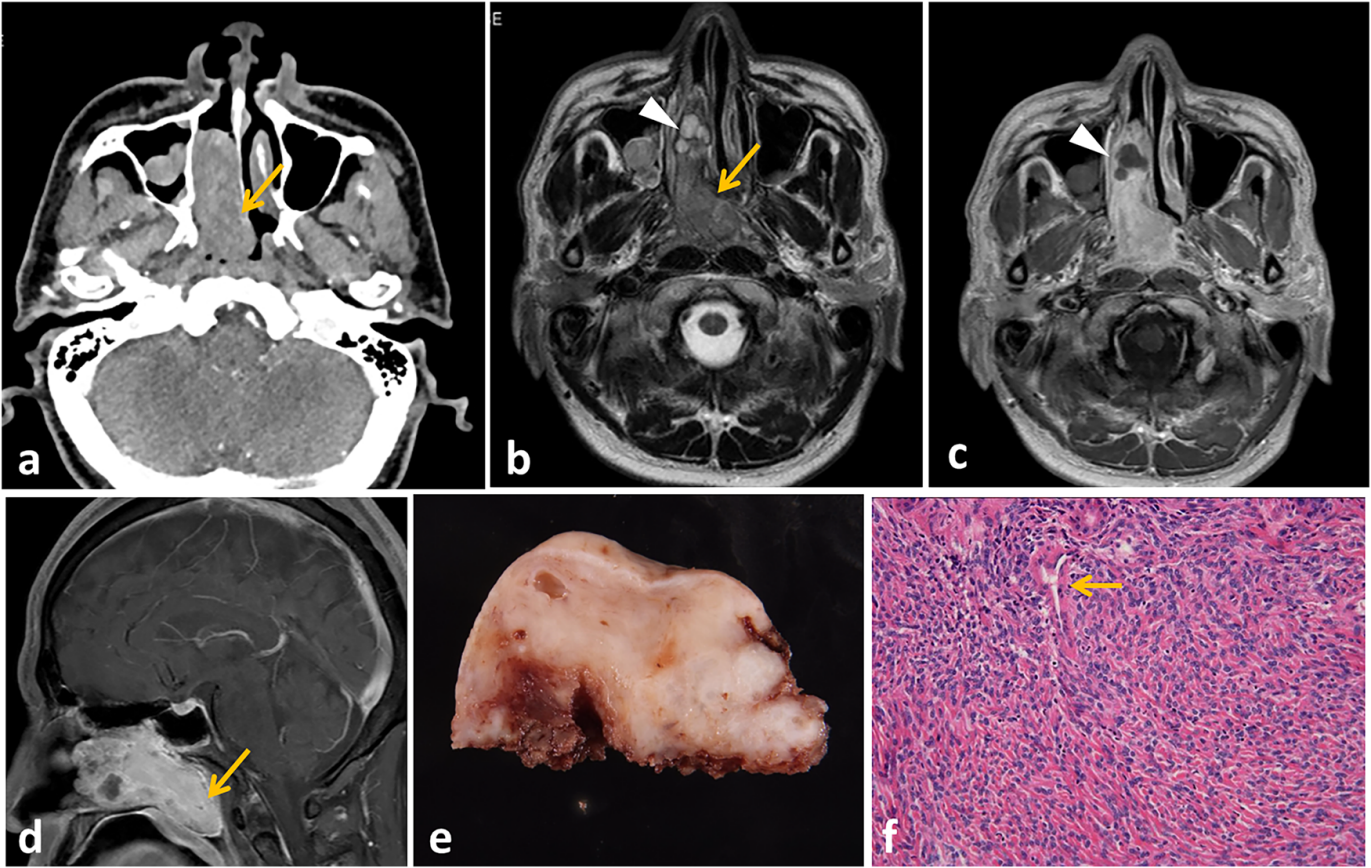
Sinosasal SFT in a 48-year-old male with a history of worsening nasal congestion for 1 year. Contrast-enhanced axial CT image (**a**) shows a well-defined homogeneously enhancing oblong mass in the right nasal cavity, extending into the nasopharynx (arrow). Axial T2- (**b**), and contrast-enhanced T1- (**c**) weighted MR images show a mass with heterogeneous isointense T2 signal (arrow), containing multiple tiny hyperintense foci that appear non-enhancing on the corresponding post-contrast image (arrowheads), suggestive of cystic changes. Sagittal contrast-enhanced T1-weighted MRI (**d**) reveals posterior extension of the mass into the nasopharynx, with marked heterogeneous enhancement (arrow). Grossly (**e**), the mass appears as a firm, well-circumscribed, lobulated solid tumor with yellowish cut section and few spaces inside. Microscopic examination (**f**) reveals spindle- to oval-shaped cells seen proliferating around staghorn-type vasculature (arrow)

Paranasal sinuses are the second most frequently affected site by SFTs in the head and neck region, accounting for a small proportion of sinonasal masses (1%). The clinical symptoms include progressive nasal obstruction and epistaxis. The majority of cases are unilateral, affecting the nasal cavity alone, followed by combined involvement of the nasal cavity and paranasal sinuses [75]. The CT radiological appearance is usually nonspecific, appearing as a lobulated homogenously enhancing mass. Large SFTs extend into orbit, nasopharynx, pterygopalatine fossa and intracranially and may also show bone thinning and remodeling or internal calcifications [76]. On MRI, these lesions appear isointense to gray matter on T1-WI and isointense or hypointense with some heterogeneity on T2-WI (Fig. 6). Sinonasal SFTs can be distinguished from other aggressive tumors by their T2 hypointense signal. The most common differential diagnoses for a sinonasal SFT are nasopharyngeal angiofibroma, inverted papilloma, hemangioma, and angiomatous polyp [77].

Nasopharyngeal angiofibroma is a unique tumor entity that predominantly affects male adolescents, typically arising in the vicinity of the sphenopalatine foramen. Characteristic features of this tumor include bone erosion and extension into the nasopharyngeal cavity. Inverted papilloma displays the characteristic convoluted cerebriform pattern on T2-WIs and post-contrast pictures. Hyperintense T2 signal with noticeable homogeneous enhancement is a characteristic of nasal hemangiomas [33]. The angiomatous polyps develop in choana and typically exhibit a strong enhancement on enhanced T1-weighted images and a hyperintense signal on T2-weighted images [76].

### Other sites

SFTs can arise in other head and neck regions such as the oral cavity, infratemporal fossa, salivary gland, thyroid gland, upper aerodigestive tract, parapharyngeal and deep cervical spaces [74, 78–80].

**Fig. 7:**
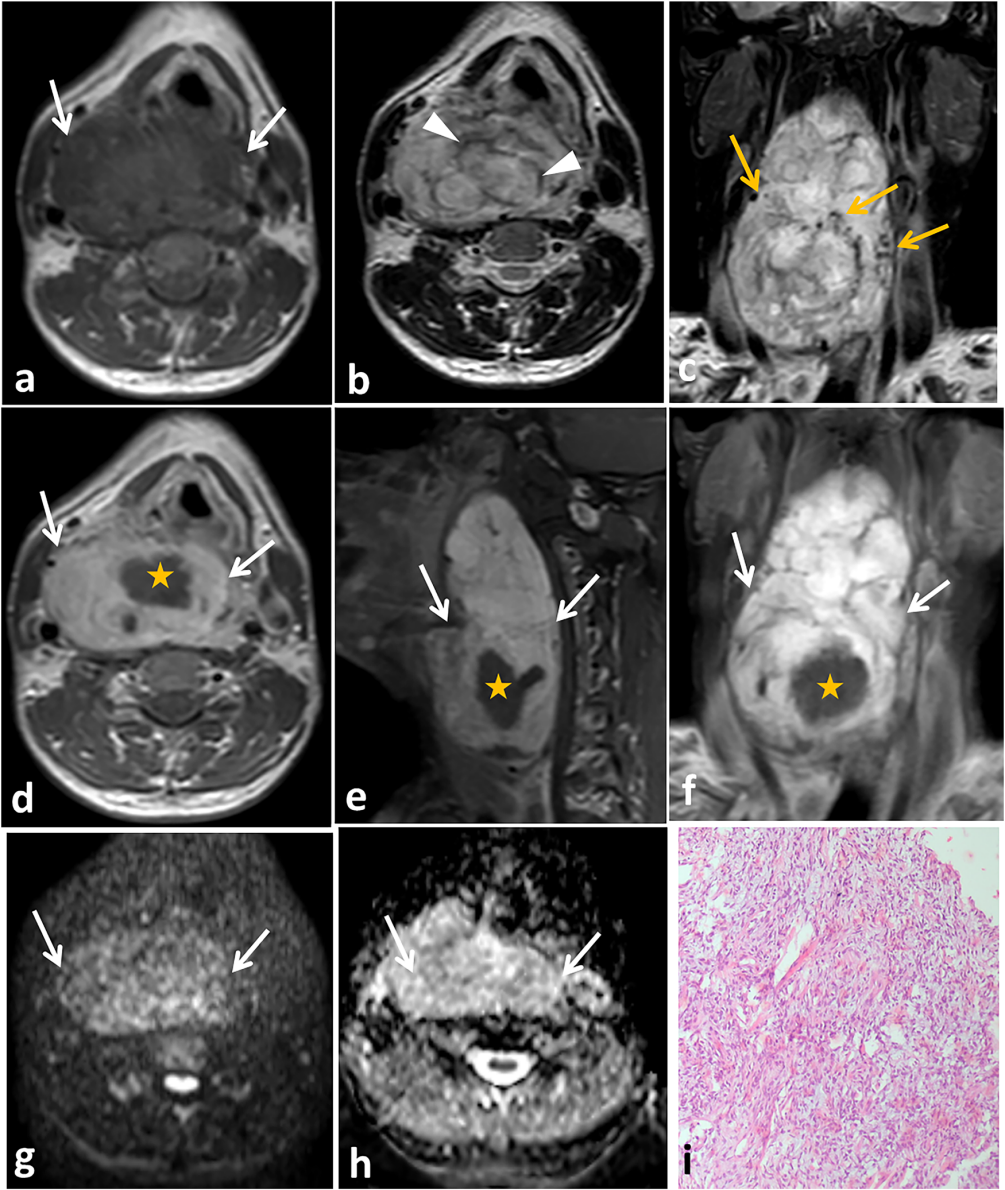
SFT of the retropharyngeal space in a 43-year-old male presented with right-side neck swelling. Non-contrast axial T1- (**a**), axial T2- (**b**) and coronal fat-suppressed T2- (**c**) weighted MRI show a large retropharyngeal soft tissue mass displaying low T1 signal intensity (white arrows) and predominantly high T2 signal intensity with linear areas of low signal intensity (arrowheads). In addition, intra- and extra-tumoral flow voids can be detected (yellow arrows). Anteriorly, the mass is seen compressing the pharyngeal mucosal space as well as the laryngeal airways, mainly on the right side. Posteriorly, it is seen compressing the prevertebral muscles with no line of separation in between. Axial (**d**), sagittal (**e**), and coronal (**f**) gadolinium-enhanced T1-weighted MR images show intense heterogeneous enhancement of the mass (arrows) with central non-enhancing areas (asterisk). Axial diffusion-weighted MR image with high b-value (**g**) and corresponding ADC map (**h**) show minimal diffusion restriction. Microscopic examination (**i**) shows spindle-shaped tumor cells with haphazard arrangement within collagenized stroma

SFTs that involve the parapharyngeal and retropharyngeal spaces reach a large size before presentation and present late with pressure symptoms, including dysphagia and dyspnea. This is due to the slowly growing painless nature of the disease. Imaging features are nonspecific. The differential diagnoses include fibrosarcoma, solitary myofibroma, metastatic malignant mesothelioma, synovial sarcoma, neurofibroma, and benign and malignant nerve sheath tumors (Fig. 7) [81, 82].

Thyroid involvement with SFT is extremely rare and can be challenging to differentiate from other thyroid neoplasms. Typically, patients present with a progressively enlarging mass or goiter. Imaging features are nonspecific and diagnosis usually depends on the histopathological evaluation and IHC [83].

## Intrathoracic

**Fig. 8:**
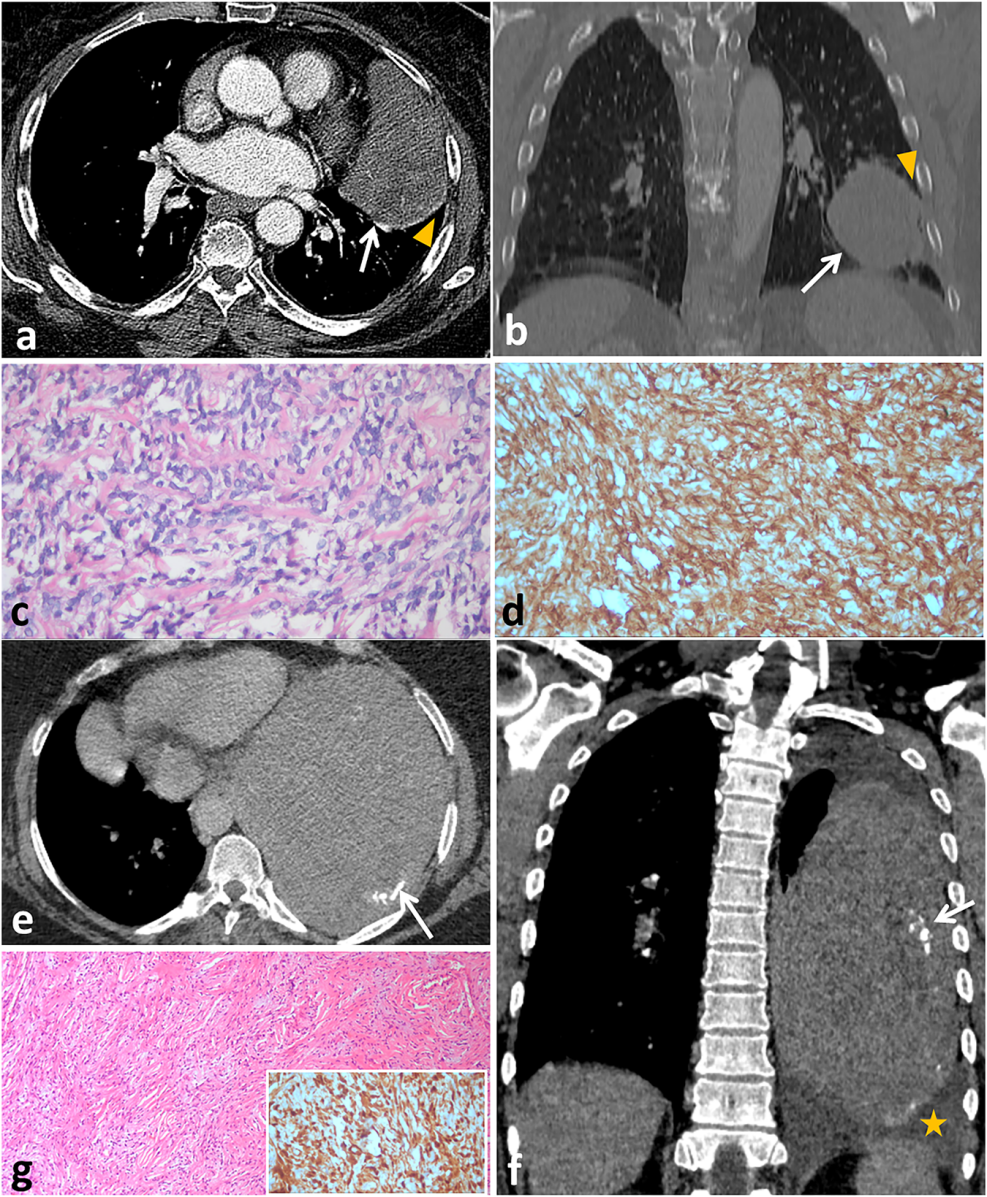
SFT of the pleura in a 76-year-old female with chest pain and cough. Axial (**a**) and coronal reformatted (**b**) contrast-enhanced CT images of the chest demonstrate a well-circumscribed, heterogeneous enhancing soft-tissue mass along the left oblique fissure (arrows). The mass has an acute angle with the pleural surface (arrowheads). Microscopic examination (high power) (**c**) and CD34 (**d**), show short poorly formed fascicles of spindle shaped tumor cells with collagen deposition. Tumor cells are diffusely positive for CD34. SFT of the pleura in a 44-year-old female presented with chest pain, cough, dyspnea, and fever for 3 months. Axial (**e**) and coronal reformatted (**f**) contrast-enhanced CT images of the chest demonstrate a large, well-circumscribed, homogeneously enhancing soft-tissue mass in the left hemithorax with peripheral calcifications (arrows). The mass has an acute angle with the pleural surface. Mild left-sided pleural effusion is also present (asterisk). Microscopic examination (**g**) and STAT 6 (inset) show haphazardly arranged short spindled tumor cells within collagenized stroma. Tumor cells are diffusely positive for STAT-6

SFTs of the pleura account for less than 5% of all pleural tumors. Despite the low incidence, their recognition has grown in recent years. [84]. Around 80% of pleural SFTs originate from the visceral pleura, while 20% come from the parietal pleura. These tumors are typically seen as distinct nodules or masses with well-defined edges, although they can also appear lobulated. Most of them possess a capsule, and a few may have a stalk that attaches them to the pleura (Fig. 8) [85, 86]. SFTs with a stalk may appear to change shape and location due to their attachment via a stalk or pedicle, features that are often better visualized on CT [85]. Approximately 50% of patients with pleural SFTs do not exhibit symptoms, and tumors are frequently discovered accidentally during chest X-rays. Nonetheless, some patients may experience cough, chest pain, and difficulty breathing due to the size of the lesions [84].

The main differential diagnosis for intrathoracic SFTs includes other neoplasms such as mesothelioma, pleural metastases, nerve sheath tumors, and soft tissue sarcomas. When distinguishing between these conditions, it is important to note that pleural mesothelioma typically presents as multiple pleural nodules or a diffuse mass enveloping a section of the lung, whereas SFTs often form a clearly defined localized mass. Recognizing the distinguishing features between benign and malignant tumors on CT scans prior to surgery is crucial for determining the appropriate clinical management. The majority of literature highlights the challenge of distinguishing between benign and malignant pleural SFTs through CT scans. However, it has been stated that malignant cases often exhibit areas of low density and irregular pleural thickening. Moreover, it has been noted that malignant SFTs typically present with a tumor diameter exceeding 10 cm and a more substantial blood supply compared to benign ones [33].

## Abdomen

### Liver

SFTs of the liver are very rare with less than 100 cases reported in the literature [87]. Hepatic SFTs could be primary or metastatic from other sites with a female predilection (ratio 1.5:1), unlike other sites. Clinical symptoms are nonspecific and often discovered incidentally [88]. Symptomatic cases typically result from mass effect, manifesting as abdominal pain, bloating, weight loss, and fatigue [87].

The radiological features of SFTs are also nonspecific. The abdominal ultrasound may display well-defined margins, heterogeneous echotexture, and occasional calcifications [89]. Contrast-enhanced CT reveals low-density masses with heterogeneous enhancement persisting into delayed phases [87]. MRI reveals tumors of low-to-intermediate T1 signal intensity and heterogeneous mixed low and high T2 signal intensity with heterogeneous post-contrast enhancement [90]. Areas of low T2 signal intensity correspond to the collagenous or fibrotic component.

The most common mimickers of hepatic SFT are hepatic hemangioma, cholangiocarcinoma, and fibrolamellar hepatocellular carcinoma. Typical hemangioma displays distinctive peripheral nodular enhancement with gradual and homogeneous centripetal progression. Cholangiocarcinoma characteristically exhibits peripheral enhancement in arterial and venous phases, with sustained enhancement during delayed phases. The presence of capsular retraction and dilated intrahepatic biliary radicles in cholangiocarcinoma and areas of low T2 signal intensity in SFTs help differentiate both tumors. Fibrolamellar hepatocellular carcinoma often occurs in young adults and features a large hypointense central scar on T2-WI [91].

### Pancreas

Pancreatic SFTS are extremely rare and only 34 cases have been reported in the English literature to date, with more female reports and equal head/body distributions. [92]. It could be primary or metastatic. Contrast-enhanced CT and MRI show enhancement through the arterial to portal phase, mimicking a non-functioning neuroendocrine tumor. Consequently, the tumors were preoperatively diagnosed as neuroendocrine neoplasms in most of the previous reports [92–95]. Nonfunctioning islet cell tumors are usually large at presentation with a predilection for the pancreatic head, and up to 90% are malignant at presentation (regional lymphadenopathy or distant metastases). Furthermore, non-enhancing portions can sometimes be found, which are mostly derived from cystic degeneration [96]. Accordingly, it should also be differentiated from solid pseudopapillary tumor of the pancreas, which is predominantly seen in young women, usually large, and contains areas of cystic change, necrosis, or hemorrhage.

### Peritoneum and mesentery

**Fig. 9:**
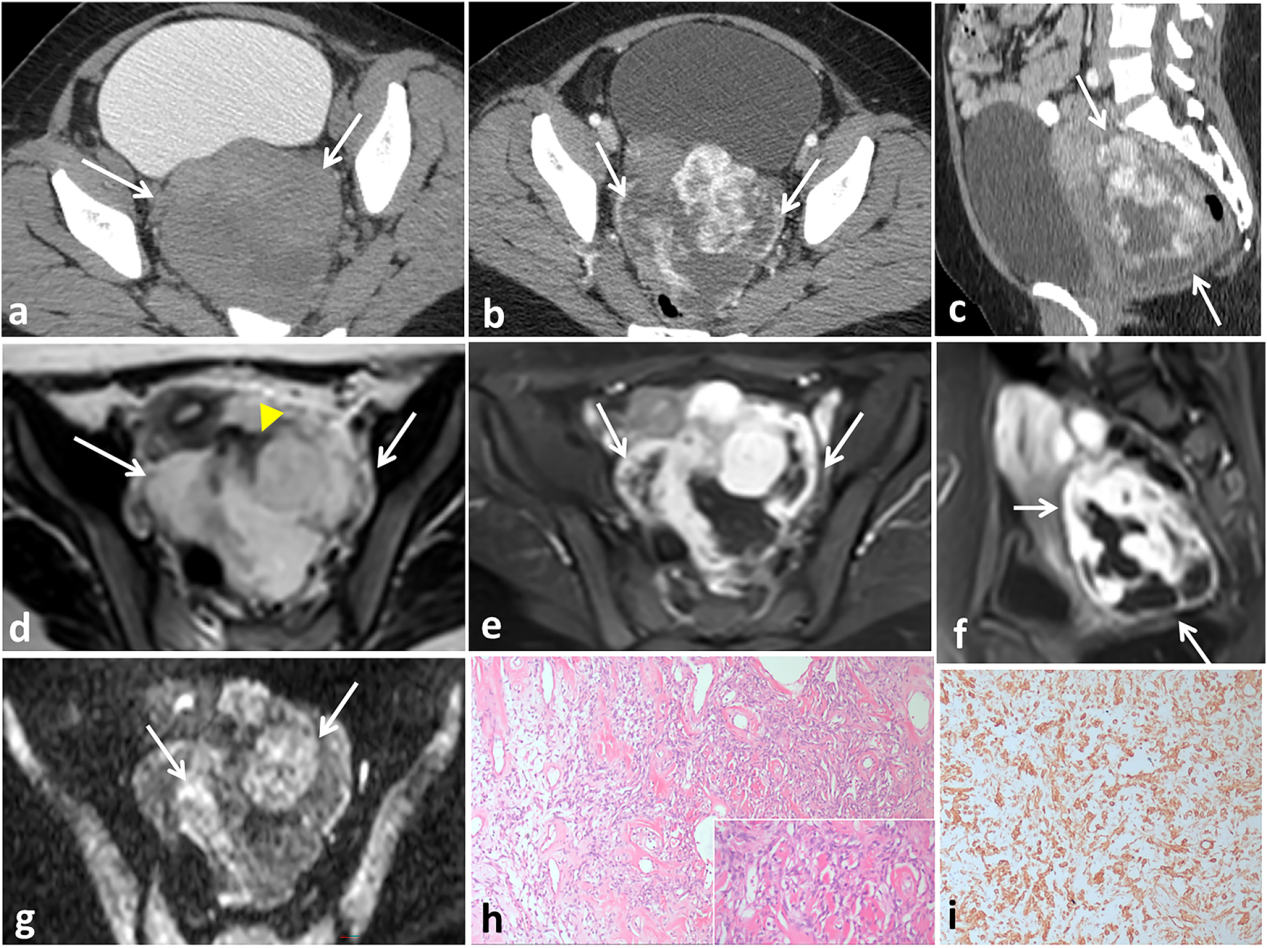
SFT of the Douglas pouch in a 24-year-old female. Axial non-enhanced CT image (**a**) with rectal and bladder contrast shows a well-defined lobulated heterogeneous density mass (arrows) in the Douglas pouch displacing the bladder anteriorly and rectum posteriorly. Axial (**b**) and sagittal reformatted (**c**) contrast-enhanced CT images show intense heterogeneous enhancement of the mass (arrows) with central non-enhancing areas, denoting necrosis or cystic degeneration. Axial T2-weighted MRI (**d**) shows the well-defined lobulated mass (arrows), which is predominantly hyperintense (white arrows) with linear areas of low signal intensity (yellow arrow), corresponding to the fibrous/collagen content. Axial (**e**) and sagittal (**f**) gadolinium-enhanced fat-suppressed T1-weighted MR images show the mass, which has intense heterogeneous enhancement (arrows) with central non-enhancing areas. The mass is seen displacing the uterus and bladder anteriorly and the rectum posteriorly. Axial diffusion-weighted MRI (**g**) with high b-value shows areas of restricted diffusion within the mass (arrows). Microscopic examination (**h**) shows haphazard proliferation of spindle tumor cells within collagenized stroma containing multiple dilated vascular spaces. CD34 (**i**) shows diffuse positive reaction

Less than 50 reported cases involving mesenteric or peritoneal SFTs are found in the English literature and have imaging manifestations identical to those of SFTs at other sites [97, 98]. Although exceedingly rare, we have encountered four cases of mesenteric and peritoneal SFTs (Figs. 2–4, 9). Preoperative differential diagnosis of mesenteric SFTs includes primary mesenteric/omental tumors (desmoid, gastrointestinal stromal tumor, malignant mesothelioma), secondary metastases (carcinoid), and mesenteric fibromatosis [99–101]. The imaging profile of desmoid tumors is characterized by well-circumscribed masses with subtle, heterogeneous enhancement, frequently associated with antecedent trauma or surgery. Gastrointestinal stromal tumors show heterogeneous enhancement with central necrosis and tumor vessels. Metastatic lymphadenopathy from neuroendocrine tumors frequently originates from small intestine carcinoid tumors, often accompanied by carcinoid syndrome. Mesenteric carcinoids exhibit poorly defined margins, delayed enhancement, mesenteric retraction, and potential bowel obstruction due to desmoplastic reaction, with frequent tumoral calcifications [99–101].

### Kidney

SFTs are rarely encountered in the kidney, with less than 110 examples of renal SFTs have been reported in the literature till date. Renal SFTs are thought to arise from the renal capsule, interstitial tissue, or peripelvic connective tissue. These tumors exhibit benign behavior, with a low propensity for recurrence or metastasis [102].

**Fig. 10:**
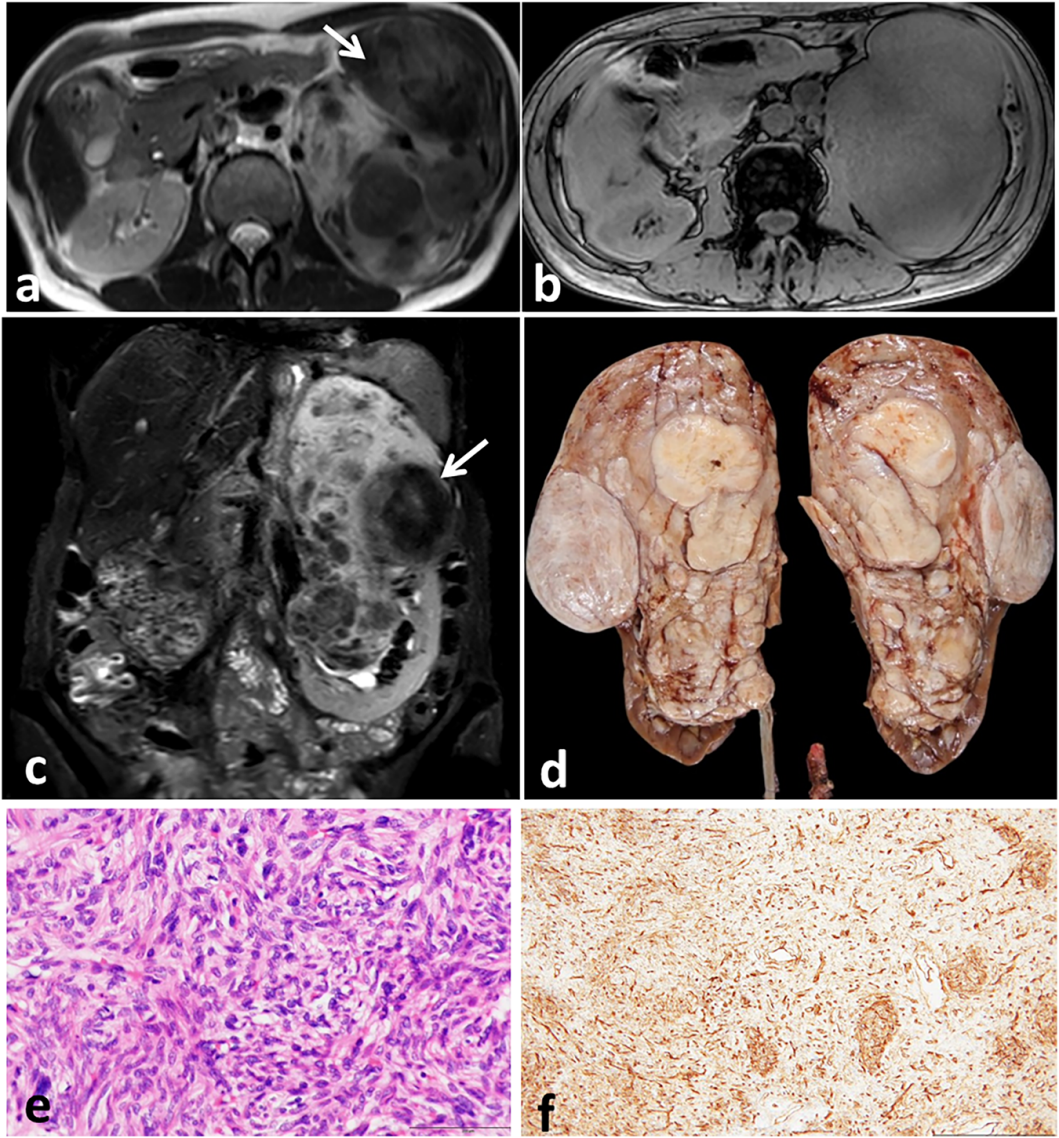
Left renal SFT in a 64-year-old female with left lumbar swelling for 1 year. Axial T2- (**a**), out of phase (**b**), and coronal T2- (**c**) weighted MRI images show a large lobulated soft tissue mass occupying most of the renal parenchyma and sparing its lower pole. The mass has T2 heterogeneous signal intensity with multiple low signal areas inside (arrows). **d** Gross examination shows a firm multinodular tumor mass with grayish white cut section occupying most of the renal parenchyma, with a remaining small rim of the renal tissue at its lower pole. Microscopic examination (**e**) shows spindle tumor cells arranged in a vague short fascicular pattern. Diffuse positive CD34 reaction is seen in tumor cells (**f**)

The differential diagnosis at imaging encompasses hypervascular renal masses such as renal cell carcinoma, oncocytoma, and metastases. Imaging findings alone cannot reliably distinguish these entities, as imaging characteristics overlap. The presence of central necrosis in large renal cell carcinomas is a characteristic that distinguishes them from SFTs. Furthermore, the low signal intensity of SFTs on T2-WI is a useful diagnostic feature that helps differentiate them from clear cell renal cell cancer (Fig. 10). However, papillary renal cell cancers typically appear hypointense on T2-weighted images, and hence cannot be differentiated from SFTs at T2-weighted imaging [103]. Most SFTs tend to be indolent; therefore, the presence of metastases raises suspicion of the more common renal cell carcinoma.

### Retroperitoneal

SFTs of the retroperitoneum are exceedingly rare, accounting for less than 30% of all SFTs [104]. Retroperitoneal SFTs tend to exhibit larger dimensions compared to those occurring in other anatomical locations [105]. Moreover, malignant and de-differentiated SFT subtypes are reportedly more prevalent in the retroperitoneum and deep soft tissues [106].

**Fig. 11:**
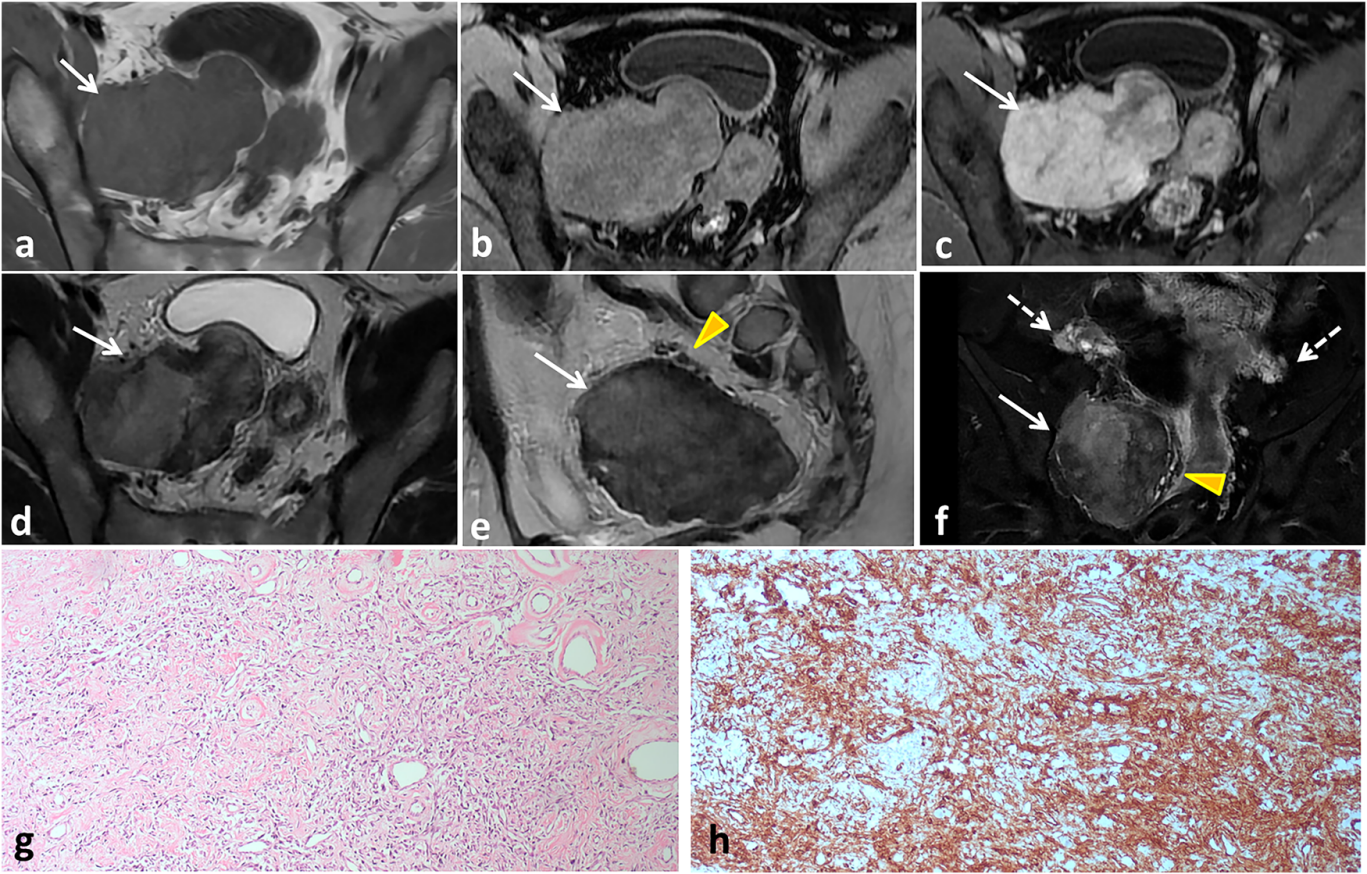
Pelvic retroperitoneal SFT in a 31-year-old female presented with lower abdominal and back pain. Axial T1- (**a**) and gadolinium-enhanced fat-suppressed T1-weighted MRI in early (**b**) and late (**c**) phases show a large right-side pelvic retroperitoneal soft tissue mass eliciting intermediate T1 signal intensity and mild heterogeneous enhancement in the early phase with marked progressive enhancement in the subsequent phase (arrows). Axial T2- (**d**), sagittal T2- (**e**) and coronal fat-suppressed T2- (**f**) weighted MRI show the mass with mixed low and intermediate signal intensity (white arrows) as well as prominent flow void at its periphery (arrowheads). Both ovaries are normal (dashed arrows). Microscopic examination (**g**) shows haphazard proliferation of spindle tumor cells within collagenized stroma containing multiple dilated vascular spaces. CD34 (**h**) shows diffusely positive reaction

At imaging, retroperitoneal SFTs commonly display heterogeneity, predominant hypervascularity, and variable degrees of necrosis and cystic degeneration (Fig. 11). The radiological differential diagnosis of retroperitoneal SFTs comprises a spectrum of mesenchymal neoplasms, including desmoid tumors, leiomyosarcomas, Gastrointestinal stromal tumors, malignant mesotheliomas, synovial sarcomas, and neurogenic tumors [33].

### Pelvis

SFTs in the pelvis commonly arise from the pelvic peritoneum and are rare entities, with clinical and imaging features similar to those of the other extrathoracic counterparts (Figs. 4, 9, 11). SFTs have been reported in the urinary bladder, prostate, seminal vesicle, uterus, ovaries, spermatic cord and ischio-anal fossa [107–110]. They usually grow slowly and are asymptomatic, unless they become large enough to cause compressive symptoms on surrounding structures. The differential diagnosis should encompass neurogenic tumors and soft tissue sarcomas [33].

## Soft tissues

SFTs originating from somatic soft tissues in different areas of the body, such as the extremities and the head, account for 10% of cases. [45]. Limited reports have discussed SFTs specifically in the extremities [111]. While these tumors are usually benign, around 10% to 20% can display aggressive characteristics [112].

**Fig. 12:**
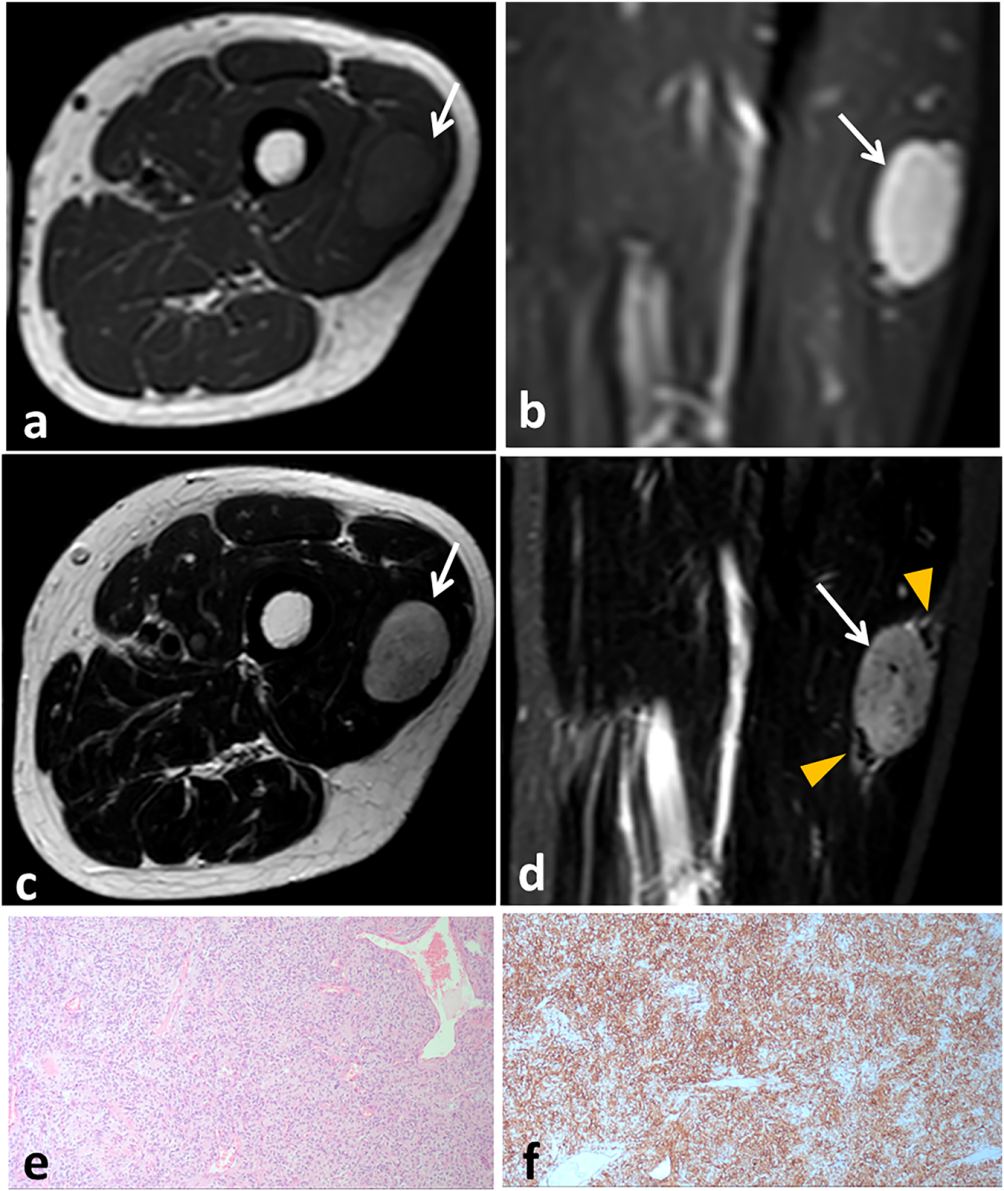
Soft tissue SFT in a 36-year-old male presented with left thigh swelling. Axial non-enhanced (**a**) and sagittal gadolinium-enhanced fat-suppressed (**b**) T1-weighted MR images show a well-defined oval soft-tissue mass intramuscular in location within the vastus lateralis muscle. The mass has T1 hypointense signal with intense heterogeneous enhancement (arrows). Axial (**c**) and sagittal fat-suppressed (**d**) T2-weighted MRI show hyperintense signal (arrows) with central non-enhancing areas and serpentine vessels along the periphery of the mass (arrowheads). Microscopic examination (**e**) show cellular tumor tissue with closely packed spindle cell proliferation and dilated staghorn-like vasculature. Tumor cells are diffusely positive for CD34 (**f**)

SFTs in the extremities have nonspecific imaging features, making them difficult to distinguish from other benign and malignant lesions. Variations in tumor appearance on CT and MRI can be influenced by the amount of collagen present, often displaying a unique mix of dark and light areas on MRI due to the presence of fibrous tissue with notable enhancement at both CT and MRI (Fig. 12) [42].

The differential diagnosis for soft tissue SFTs is extensive, but key considerations include desmoid tumors, nodular fasciitis, and dermatofibrosarcoma protuberans. Desmoid tumors have distinct imaging characteristics, appearing isodense to muscle on CT with homogeneous enhancement and possible calcifications. MRI reveals isointense to hypointense signals on T1- and T2-weighted images, with uniform enhancement on contrast-enhanced images [113, 114]. Nodular fasciitis often resolves spontaneously and rarely recurs post-excision [113]. Notably, 35% of soft tissue SFTs feature a vascular pedicle, aiding in differential diagnosis.

## Bone

Intramedullary SFTs of osseous origin are exceptionally rare, with a predilection for spinal involvement, whereas long bone involvement is exceedingly uncommon [115, 116]. Patients typically present with localized pain, which may radiate due to neural compression. Radiological findings reveal lytic lesions with narrow, non-sclerotic transition zones and potential cortical breakthrough, which are indistinguishable from those of other intermediate-aggressive bone neoplasms.

Local recurrence may stem from incomplete surgical excision rather than atypical histological characteristics. However, even with complete resection, SFTs of bone can recur locally or metastasize [116, 117].

## Natural history and management

The primary effective therapeutic modality for SFTs involves surgical intervention. Optimal treatment for pleural SFTs involves complete surgical resection with negative margins, ensuring superior outcomes and prolonged survival [118]. However, the propensity for recurrence in localized pleural SFTs (10–30% after R0 resection) underscores the imperative for vigilant long-term surveillance and follow-up care [119].

SFTs benefit from radiation therapy as neoadjuvant or adjuvant treatment for high-risk features, complementing surgical management [120, 121].

Given the limitations of surgical intervention in advanced or metastatic cases, systemic therapy with sarcoma-directed chemotherapy agents represents a promising avenue for advanced SFT treatment [122].

It is worth noting that the therapeutic landscape of SFTs is expanding to include anti-angiogenic agents, which have demonstrated encouraging efficacy in overcoming chemotherapy resistance [123, 124]. Pazopanib is recommended as first-line treatment for typical and malignant SFTs, demonstrating significant efficacy [125]. In addition, immunotherapy offers new hope for SFT treatment by boosting T-cell response [27].

## Conclusion

SFTs are rare, hypervascular neoplasms exhibiting variable locations, potential aggressiveness, and metastatic capability. SFTs are typically hypervascular masses with characteristic intense, heterogeneous enhancement during the arterial phase of dynamic contrast-enhanced CT or MRI and progressive enhancement in later phases. A frequent and unifying feature of SFT at MRI is the presence of low signal intensity foci on T1- and T2-weighted images, corresponding to the fibrous and collagenous content. SFTs exhibit diverse clinical behaviors, necessitating comprehensive diagnostic evaluation and long-term surveillance following complete resection.

## Data Availability

Data are available upon request.

## Abbreviations

CNS: Central nervous system
CT: Computed tomography
HPC: Hemangiopericytoma
IHC: Immunohistochemistry
MRI: Magnetic resonance imaging
PET: Positron emission tomography
SFT: Solitary fibrous tumors
STAT6: Signal transducer and activator of transcription 6
WHO: World Health Organization
WI: Weighted image
